# The Effects of Simulated Microgravity on Macrophage Phenotype

**DOI:** 10.3390/biomedicines9091205

**Published:** 2021-09-12

**Authors:** Christopher Ludtka, Erika Moore, Josephine B. Allen

**Affiliations:** 1 J. Crayton Pruitt Family Department of Biomedical Engineering, University of Florida, Gainesville, FL 32611, USA; cludtka@ufl.edu; 2 Materials Science and Engineering, University of Florida, Gainesville, FL 32611, USA; moore.erika@ufl.edu

**Keywords:** macrophage, phenotype, microgravity, immune, pro-inflammation, classically activated, alternatively activated

## Abstract

The effects of spaceflight, including prolonged exposure to microgravity, can have significant effects on the immune system and human health. Altered immune cell function can lead to adverse health events, though precisely how and to what extent a microgravity environment impacts these cells remains uncertain. Macrophages, a key immune cell, effect the inflammatory response as well as tissue remodeling and repair. Specifically, macrophage function can be dictated by phenotype that can exist between spectrums of M0 macrophage: the classically activated, pro-inflammatory M1, and the alternatively activated, pro-healing M2 phenotypes. This work assesses the effects of simulated microgravity via clinorotation on M0, M1, and M2 macrophage phenotypes. We focus on phenotypic, inflammatory, and angiogenic gene and protein expression. Our results show that across all three phenotypes, microgravity results in a decrease in TNF-α expression and an increase in IL-12 and VEGF expression. IL-10 was also significantly increased in M1 and M2, but not M0 macrophages. The phenotypic cytokine expression profiles observed may be related to specific gravisensitive signal transduction pathways previously implicated in microgravity regulation of macrophage gene and protein expression. Our results highlight the far-reaching effects that simulated microgravity has on macrophage function and provides insight into macrophage phenotypic function in microgravity.

## 1. Introduction

Understanding the effects of the space environment on cellular behavior is critical when considering human health in the context of spaceflight [1,2]. In particular, the effects of altered gravity on human, animal, and plant cells are frequently reported to have demonstrable effects on cellular function and expression profiles [3,4]. Among the cell types being evaluated, immune cells are of particular interest due to their critical role in pathology and human health [5,6]. There are well documented reports of humans experiencing viral shedding while in space, which further motivates studies to assess the effect of space flight on immune cells [7,8,9]. Macrophage immune cells are present in the majority of tissues throughout the body and are involved in immunological functions as well as tissue remodeling. Phenotypic differences in macrophages inform their function. Macrophage phenotypes consist of a non-polarized M0 state, a pro-inflammatory, classically activated M1 state, and a pro-healing, alternatively activated M2 state. Each of the macrophage phenotypes vary in their gene expression, surface marker expression, and cytokine secretion profiles. Published data on the effect of altered gravity on macrophages have not extensively considered the role of macrophage phenotypes. Macrophage microgravity studies in general have shown variable results and are relatively limited [10,11]. Overall, macrophages demonstrate many sensitivities to changes in gravity, both in their short- and long-term responses. Reports have shown that microgravity (real or simulated) induces changes in macrophage metabolism, signal transduction, proliferation, differentiation, cytokine secretion, cytoskeletal structure, gross morphology, locomotion, gene expression, and inflammatory response [12,13,14]. Changes in any of these cellular processes can contribute to an altered immune response while in space. Given the important role of macrophage behavior and function in human health, we investigate the effects of a simulated microgravity environment across M0, M1, and M2 macrophage phenotypes.

To assess the effect of simulated microgravity across major macrophage phenotypes, we evaluate the expression of key macrophage phenotypic markers, as well as proteins associated with inflammation, angiogenesis, and cell structure. As an antigen-presenting cell and phagocyte, the macrophage is a critical cell within the immune response. Additionally, it has a role in tissue remodeling and vascularization through interactions with endothelial cells [15,16]. For instance, M2 phenotype macrophages have been reported to demonstrate a major role in reorganization of the vasculature [17]. It has been hypothesized that macrophages mediate vascular organization through vascular endothelial growth factor (VEGF) secretion, as the absence of macrophages resulted in irregular vessel formation. Meanwhile, M1 phenotype macrophages produce greater amounts of nitric oxide, mediating the immunological purging of mycobacteria and parasites [18]. Previous literature evaluating macrophages in the context of microgravity has largely neglected phenotypic differences, even though they play a major role in macrophage functionality. The majority of studies use pro-M1 stimulatory cytokines following microgravity to induce macrophage cytokine expression and secretion for analysis and rarely directly compare expression to a baseline, non-stimulated M0 state [5,19,20,21,22]. Of the very limited studies that have investigated macrophages using pro-M2 factors, stimulation is again only administered during or after exposure to microgravity [12,23]. As there have been reports that microgravity can inhibit macrophage polarization [12], this may affect the expression levels seen if macrophages are being impeded from achieving the desired M1 and M2 phenotypes. Nevertheless, to offer insight into how phenotypes can be altered in microgravity, it is important to understand how microgravity affects the stability of these phenotypically distinct macrophage populations, their polarization, and their downstream function.

The gold standard for microgravity studies would be to study macrophages in real microgravity during and after space flight, however this is logistically challenging and can be cost prohibitive. Other opportunities to expose cells to real microgravity include suborbital flight/sounding rocket and parabolic flight modalities, however, these face many of the same challenges. Alternatives to real microgravity include a variety of methods commonly used to expose cultured cells to simulated microgravity, including several benchtop methods, such as clinorotation and random position machines [10,11]. In this work, we utilize a rotary cell culture system (RCCS), specifically the rotating wall vessel (RWV) originally developed by the National Aeronautics and Space Administration, to expose phenotypically divergent macrophages to simulated microgravity and assess the acute biological effects. It is important to note that simulated microgravity methods are limited in their direct comparability to real microgravity, as, for instance with the RWV system, simulated microgravity is achieved via a sedimentation velocity where gravity is counterbalanced by hydrodynamic shear, centrifugal, and Coriolis forces [24]. As such, simulation methods do not perfectly reflect the physical conditions of real microgravity experienced in spaceflight.

In this work, we compare macrophages experiencing simulated microgravity to normal gravity controls for each of the three major phenotypes. Using standard methods, the macrophages are phenotypically stimulated prior to simulated microgravity exposure, allowing us to report the effect of this altered environment on each distinct population. Herein we report phenotype-dependent changes, including phenotypic gene expression, inflammatory cytokine production, and the production of angiogenic growth factors. We report the affect that simulated microgravity has on macrophage phenotype and provide insight into macrophage phenotypic function in this unique environment. This work provides a deeper understanding of how the space environment may be altering immune cells in a phenotype-dependent manner.

## 2. Materials and Methods

### 2.1. Cell Culture and Simulated Microgravity

RAW 264.7 cells were acquired from ATCC (Manassas, VA, USA). These cells are a murine macrophage cell line derived from adult male BALB/c mice. The RAW 264.7 cells were cultured at 37 °C and 5% CO_2_ using Dulbecco’s Modified Eagle Medium (DMEM) supplemented with 10% v/v fetal bovine serum (FBS) and 1% v/v penicillin/streptomycin (P/S) all from Corning (Corning, NY, USA). As macrophages are an adherent cell type, the cells were attached to microcarrier beads prior to clinorotation, which is recommended for anchorage-dependent cells [12,19,25] To achieve this experimental set-up the macrophages were subsequently mixed and cultured with fibronectin coated (25 ng/mL) Cytodex 1 microcarrier beads (Cytiva, Marlborough, MA, USA) at a previously optimized cell seeding density of 150,000 cells (M0, M2 phenotypes) or 300,000 cells (M1 phenotype) using 2.4 mg (dry weight) of beads for each individual sample. Cell attachment took place over 2 days in a Corning Ultra-Low Attachment well-plate at 37 °C and 5% CO_2_. Simultaneous to attachment, cells were divided into three groups and treated with either the basal DMEM previously described (10% FBS, 1% P/S) to remain in the M0 phenotype, or with polarization media to induced M1 and M2 phenotypes. M1 media was basal DMEM supplemented with 10 ng/mL of IFN-γ (Novus Biologicals, Centennial, CO, USA) and 100 ng/mL of LPS (Santa Cruz Biotech, Dallas, TX, USA). M2 media was basal DMEM supplement with 20 ng/mL of IL-4 (BioLegend, San Diego, CA, USA). Following 2-day attachment and polarization, the macrophage populations (M0, M1 and M2) were cultured in either normal gravity or simulated microgravity in basal DMEM media without any phenotype driving supplements. Simulated microgravity was achieved using a NASA inspired and developed Rotating Wall Vessel (RWV) Cell Culture System (Synthecon, Houston, TX, USA).

**Figure 1:**
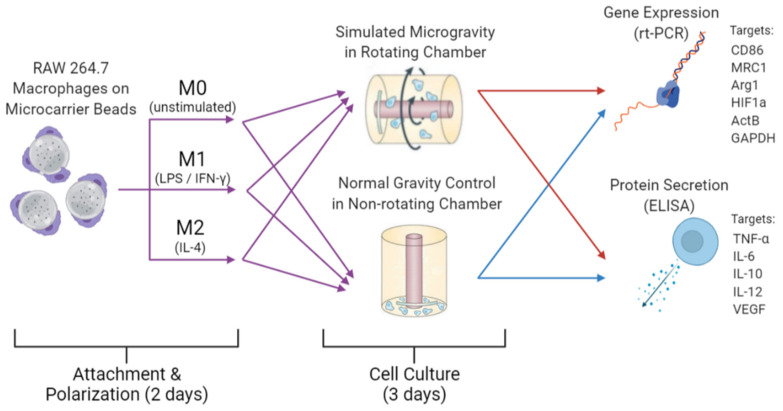
Visual schematic of experimental design. Purple arrows indicate preparations of cells prior to RWV culture for all three phenotypes. Red and blue arrows indicate simulated µG samples and control 1G samples, respectively, were analyzed via RT-PCR and ELISA subsequent to 3 days of cell culture under their respective conditions. Created with BioRender.com.

Briefly, cells on beads were transferred to a RWV culture vessel that was either attached to the RWV rotating base and rotated at a speed of 14 rpm to achieve simulated microgravity, or the chamber was not rotated and served as 1G (normal gravity) controls. The macrophages were cultured in either normal gravity (1G) or simulated microgravity (µG) for 3 days at 37 °C and 5% CO_2_. After 3 days, the macrophages on beads were harvested from each of the RWV chambers and the resulting media stored at −80 °C for subsequent gene and protein analysis. A schematic describing the experimental design is shown in Figure 1.

### 2.2. Protein Production Analysis

Secreted proteins in culture media from simulated microgravity and normal gravity macrophage cultures were assessed via enzyme linked immunosorbent assay (ELISA). The production and secretion of inflammatory cytokines—specifically tumor necrosis factor alpha (TNF-α), interleukin 6 (IL-6), interleukin 10 (IL-10), and interleukin 12 (IL-12)—was quantified using a Murine Custom Multi-Analyte ELISArray (Qiagen, Germantown, MD, USA). Angiogenic vascular endothelial growth factor (VEGF) secretion was quantified using a VEGF ELISA (RayBiotech, Peachtree Corners, GA, USA). In all cases, ELISA was run following the manufacturer’s protocol. Absorbance readings were taken at 450 nm and 570 nm using a BioTek Synergy H1 Microplate Reader (BioTek Instruments, Winooski, VT, USA). Protein concentrations were obtained from a standard curve and were normalized to DNA concentration of the cells in each culture chamber. Normalizing DNA concentration was obtained from macrophages on beads that were lysed with Triton-Χ-100 (Thermo Fisher Scientific, Waltham, MA, USA) for 10 min at room temperature followed by quantification using a PicoGreen Quant-iT dsDNA Assay Kit according to the manufacturer’s protocol (Invitrogen, Carlsbad, CA, USA). Protein concentration data (measured as pg/mL) were normalized to DNA concentration (ng/mL) for each respective sample. Data are presented as mean ± standard deviation. A *p*-value < 0.05 was considered statistically significant, using the unpaired Student’s *t*-test.

### 2.3. Gene Expression Analysis

RNA was isolated and purified from the macrophage populations grown in simulated microgravity as well as normal controls using a Quick-RNA Microprep Kit (Zymo, Irvine, GA, USA) according to the manufacturer’s protocol, including optional DNAse treatment. RNA yield and purity was quantified using a Thermo Scientific NanoDrop One^C^ Microvolume UV-Vis Spectrophotometer (Thermo Fisher Scientific, Waltham, MA, USA). Purified RNA was reverse transcribed into cDNA (Bio-Rad iScript^TM^ Reverse Transcript Supermix) following the manufacturer’s temperature protocol using a Bio-Rad C1000 Touch^TM^ Thermal Cycler (Bio-Rad, Hercules, CA, USA). Resulting cDNA was then combined with RNAse/DNAse-free water (Bio-Rad iTaq^TM^ Universal Sybr^®^ Green Supermix), and respective primers according to the associated Sybr^®^ protocol (Bio-Rad, Hercules, CA, USA). Several macrophage phenotypic marker genes were assessed; cluster of differentiation 86 (CD86) is a marker of the M1 phenotype, and mannose receptor (MRC1) and aginase-1 (Arg1) are markers of the M2 phenotype. Beta actin (ActB) is a marker for actin as a cytoskeletal element. Finally, GAPDH is a housekeeping gene used to normalize the data. Primer oligonucleotides were obtained from OriGene (Rockville, MD, USA) and Integrated DNA Technologies (Newark, NJ, USA) using commercially available sequences. Forward and reverse primers sequences are listed in Table 1. PCR was completed using the Two-Step Amplification protocol. Each sample was run in three technical replicates. PCR data were evaluated using the comparative C_t_ method based on the reference gene GAPDH and relative to 1G controls. Data are presented as mean ± standard deviation. A *p*-value < 0.05 was considered statistically significant.

### 2.4. Statistical Analysis

Statistical analysis was conducted in GraphPad Prism 9 (v9.2.0, GraphPad Software, Inc., San Diego, CA, USA). Numerical data are reported as mean ± standard deviation (SD). Statistical significance was calculated using unpaired Student’s *t*-test, with a value of *p* < 0.05 being considered statistically significant.

## 3. Results

### 3.1. Cell Viability

**Figure 2:**
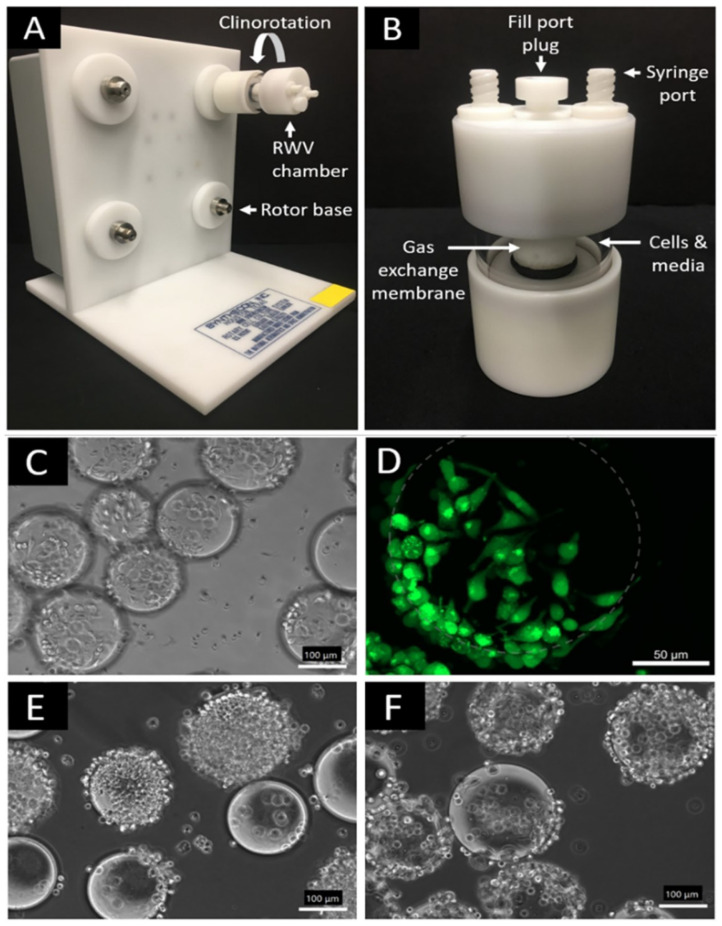
Rotary Cell Culture System Rotating Wall Vessel (RWV) bioreactor and representative microscopy images of RAW 264.7 macrophage cells on microcarrier beads. (**A**) RWV bioreactor base; (**B**) RWV bioreactor detachable vessel chamber; (**C**) M2 macrophage phenotype after 2-day attachment and polarization period (scale bar 100 μm); (**D**) Calcein-stained M2 macrophage cells after 2-day attachment period; dotted grey circle to approximate underlying bead for visual clarity (scale bar 50 μm); (**E**) 1G normal gravity control M2 macrophages (scale bar 100 μm); and (**F**) simulated µG M2 macrophages after 3-day period in RWV chambers (scale bar 100 μm).

Utilizing a rotating wall vessel (RWV) bioreactor as shown in Figure 2A,B, we successfully cultured M0, M1, and M2 macrophages in simulated microgravity. Microscopy images show that RAW 264.7 macrophage-like cells were successfully attached to microcarrier beads prior to simulated microgravity (µG) or normal (1G) control culture. (Figure 2C–F). As an adherent cell type, attachment to microcarrier beads is necessary for rotary cell culture and has commonly been reported in the context of macrophage simulated microgravity studies as well as with other adherent cell types [12,19,25,26,27]. No qualitative difference could be seen visually in cell density or cell attachment between simulated µG and 1G control samples following 3-day culture. In addition, based upon calcein staining, the cells inserted into the RWV bioreactor were alive and healthy. Following simulated microgravity culture, we did not observe a significant increase in floating, detached, or dead cells that would compromise our analysis.

### 3.2. Simulated Microgravity Effect on M0 (Non-Polarized) Macrophage Phenotype

#### 3.2.1. M0 Protein Production and Secretion

**Figure 3:**
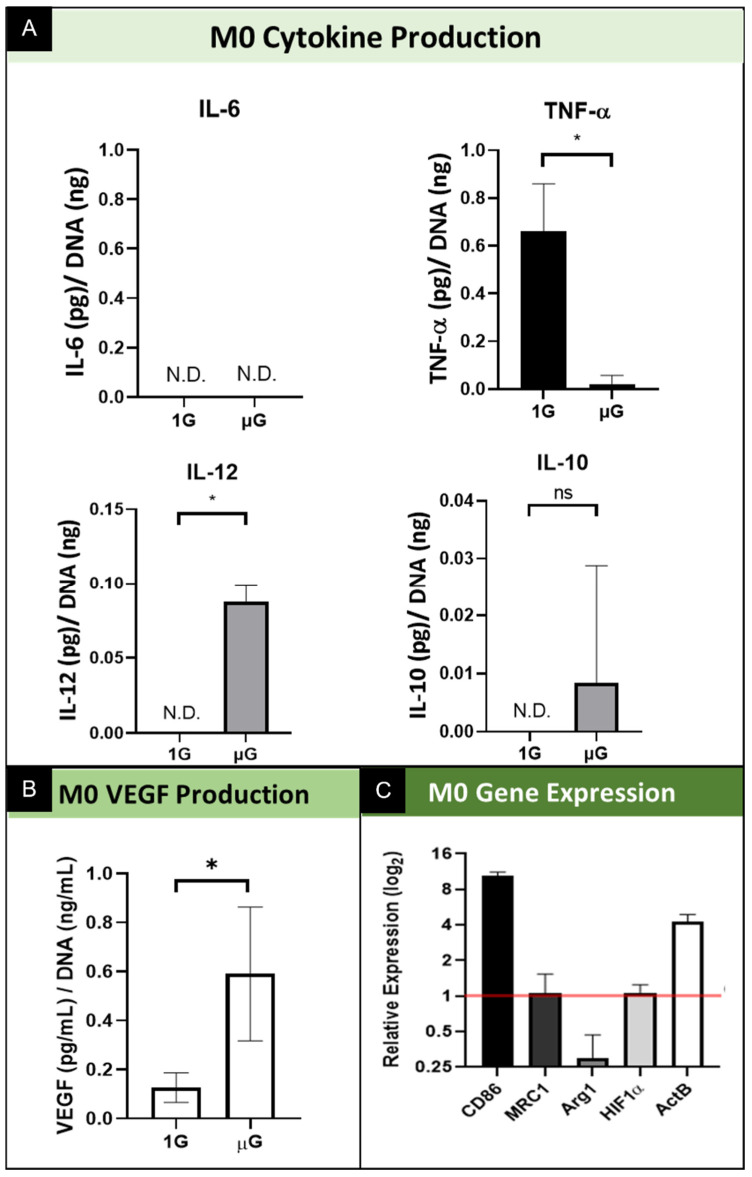
Summary of the effect of simulated microgravity on M0 macrophage protein and gene expression. (**A**) Quantification of M0 macrophage secretion of inflammatory cytokines while cultured in both normal gravity (1G) and simulated microgravity (µG) for IL-6, TNF-α, IL-10, and IL-12. Data normalized to DNA concentration and are presented as Mean +/− SD, *n* = 3. N.D. indicates the concentration did not reach the threshold for the assay and thus is Not Detected. Significance determined by unpaired Student’s *t*-test; * denotes *p* < 0.05; ns denotes non-significant. (**B**) Quantification of VEGF secretion by M0 macrophages cultured in normal gravity (1G) and simulated microgravity (µG). Data normalized to DNA concentration and are presented as Mean +/− SD, *n* = 3; Significance determined by unpaired Student’s *t*-test; * denotes *p* < 0.05. (**C**) Gene expression of M0 phenotype macrophages for M1 specific (CD86) and M2 specific (MRC1 and Arg1) genes, transcription factor, HIF1α, and structural gene ActB. Data displayed as relative expression of simulated µG samples compared to 1G controls (red line).

M0 macrophages typically shift from a M0 phenotype into either an M1 or M2 phenotype. This is the basal phenotype of macrophages, with those stimulated into either a M1 or M2 phenotype eventually reverting to the M0 phenotype within 12 days of cessation of cytokine exposure in vitro [28]. Production of inflammatory cytokines was evaluated for M0 macrophages, comparing simulated µG samples to 1G normal gravity controls. (Figure 3A) In the non-polarized macrophages of the M0 phenotype, the concentration of inflammatory cytokine IL-6 fell below the detection limit of the ELISA array for both macrophages cultured in simulated µG as well as 1G samples; as such it is shown graphically as not detected (N.D.). In contrast, the concentration of secreted TNF-α by non-polarized M0 macrophages decreased significantly when cultured for 3 days in simulated µG. The secretion of IL-12 significantly increased from undetectable levels in normal gravity (1G) to a significant increase in simulated µG. Finally, M0 macrophages showed variable levels of secreted cytokine IL-10 in simulated µG relative to the undetected level secreted in normal gravity, however this was not a statically significant change between these two conditions. As it relates to the angiogenic functional potential of M0 macrophages, as indicated by the secretion of vascular endothelial growth factor (VEGF), the data show that for the M0 macrophages culture in simulated µG, there was increased VEGF secretion relative to normal 1G control. (Figure 3B) These results demonstrate that in the absence of any soluble factors, simulated microgravity promotes M0 macrophages to secrete pro-inflammatory cytokines at variable levels. Interestingly, we see a significant shift downward in TNF-α cytokine secretion, which is not necessarily expected but is consistent with previous reports for macrophages [5,19,20,21].

#### 3.2.2. M0 Gene Expression

Quantitative real-time PCR was conducted to assess the effects of simulated microgravity on macrophage gene expression for phenotype and functional specific genes. Several macrophage phenotypic marker genes were assessed; cluster of differentiation 86 (CD86) is a marker of the M1 phenotype, and MRC1 and Arg1 are markers of the M2 phenotype. Beta actin (β-actin; ActB) was assessed to macrophage cytoskeletal actin. We also include the transcription factor HIF1α as a regulatory element involved in VEGF signaling. GAPDH was used as a reference gene for PCR data analysis, and relative expression was calculated using the comparative C_t_ method. For M0 macrophages, assessment of the M1 specific gene CD86 is significantly upregulated in simulated microgravity relative to normal gravity control. (Figure 3C) This contrasts with the gene expression of M2 specific genes, which show no change in MRC1 and a reduction in gene expression for Arg1. As it relates to the structural gene, β-actin, we show gene expression significantly upregulated in M0 macrophages cultured in simulated microgravity. Lastly, we show no change in the level of expression for transcription factor HIF1α. These results demonstrate simulated microgravity promotes alterations in M0 macrophages towards the M1 pro-inflammatory state via upregulation of M1 specific gene expression. In addition, these data indicate that simulated microgravity promotes changes in elements of cell structure, as evidenced by the increase in structural protein gene expression. Lastly, these data indicate that although VEGF secretion was upregulated by microgravity, we do not see the corresponding increase in the expression of this VEGF regulator.

### 3.3. Simulated Microgravity Effect on M1 (Pro-Inflammatory) Macrophage Phenotype

#### 3.3.1. M1 Macrophage Protein Production and Secretion

**Figure 4:**
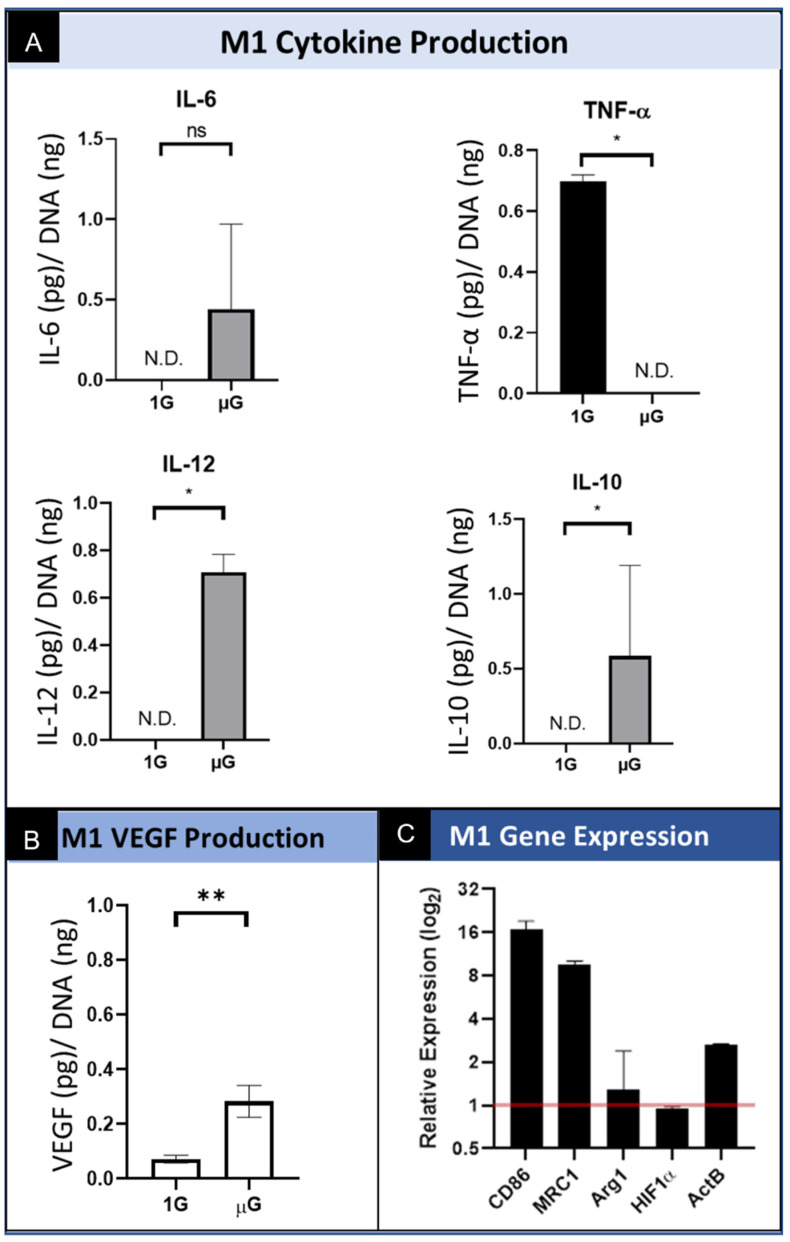
Summary of the effect of simulated microgravity on M1 macrophage protein and gene expression. (**A**) Quantification of M1 macrophage secretion of inflammatory cytokines while cultured in both normal gravity (1G) and simulated microgravity (µG) for IL-6, TNF-α, IL-10, and IL-12. Data normalized to DNA concentration and are presented as Mean +/− SD, *n* = 3. N.D. indicates Table 1 macrophages cultured in normal gravity (1G) and simulated microgravity (µG). Data normalized to DNA concentration and are presented as Mean +/− SD, *n* = 3; Significance determined by unpaired Student’s *t*-test; * denotes *p* < 0.05; ns denotes non-significant. (**B**) Quantification of VEGF secretion by M1 macrophages cultured in normal gravity (1G) and simulated microgravity (µG). Data normalized to DNA concentration and are presented as Mean +/− SD, *n* = 3; Significance determined by unpaired Student’s *t*-test; * denotes *p* < 0.05; ** denotes *p* < 0.01. (**C**) Gene expression of M1 phenotype macrophages for M1 specific (CD86) and M2 specific (MRC1 and Arg1) genes, transcription factor, HIF1α, and structural gene ActB. Data displayed as relative expression of simulated µG samples compared to 1G controls (red line).

Macrophages underwent phenotypic differentiation following stimulation with interferon gamma (IFN-γ) and lipopolysaccharide (LPS) into “classically activated” M1 macrophages which are pro-inflammatory [15,29]. The levels of M1 macrophage secreted IL-6 are variable in simulated µG, and although it is increased on average, there is no significant difference in the level of secreted IL-6 relative to cells cultured in normal gravity. (Figure 4A) We also show that M1 polarized macrophages secreted significantly less TNF-α in simulated µG compared to the normal gravity 1G control. Interestingly, the level of IL-10 and IL-12 secreted by M1-polarized macrophages cultured in simulated µG was significantly increased over the normal 1G control conditions. Regarding the angiogenic potential of M1 macrophages, the data show that there was a significant increase in VEGF secretion under simulated microgravity relative to normal gravity. (Figure 4B) These results demonstrate an unexpected trend in the upregulation of both a pro-inflammatory cytokine (IL-12) and an anti-inflammatory cytokine (IL-10). The upregulation of both IL-12 and IL-10 suggests the presence of multiple phenotypes during simulated microgravity culture. These data also show that the M1 phenotype may provide for angiogenic stimulation when exposed to this altered environmental condition, as evidenced by the upregulation of the angiogenic growth factor VEGF.

#### 3.3.2. M1 Macrophage Gene Expression

M1 macrophages demonstrated significantly increased CD86 expression in simulated µG compared to 1G controls. Notably, MRC1 expression was also elevated in simulated µG, though considered a M2 marker. The other M2 phenotypic marker, Arg1, appeared to be approximately equal between the simulated µG and 1G conditions. Like other macrophage phenotypes, HIF1α demonstrated seemingly no response to simulated µG. ActB was seen to be upregulated as well. (Figure 4C). These results demonstrate unexpected trends we saw previously in the secretion of cytokines. The M1 macrophages under conditions of simulated microgravity show significant upregulation of M1 genes (CD86) as well as upregulated M2 genes (MRC1). Lastly, consistent with earlier results, these data indicate that although VEGF secretion was upregulated in M1 macrophages by simulated microgravity, we do not see the corresponding increase in the expression of HIF1α.

### 3.4. Simulated Microgravity Effect on M2 (Pro-Healing) Macrophage Phenotype

#### 3.4.1. M2 Macrophage Protein Production and Secretion

**Figure 5:**
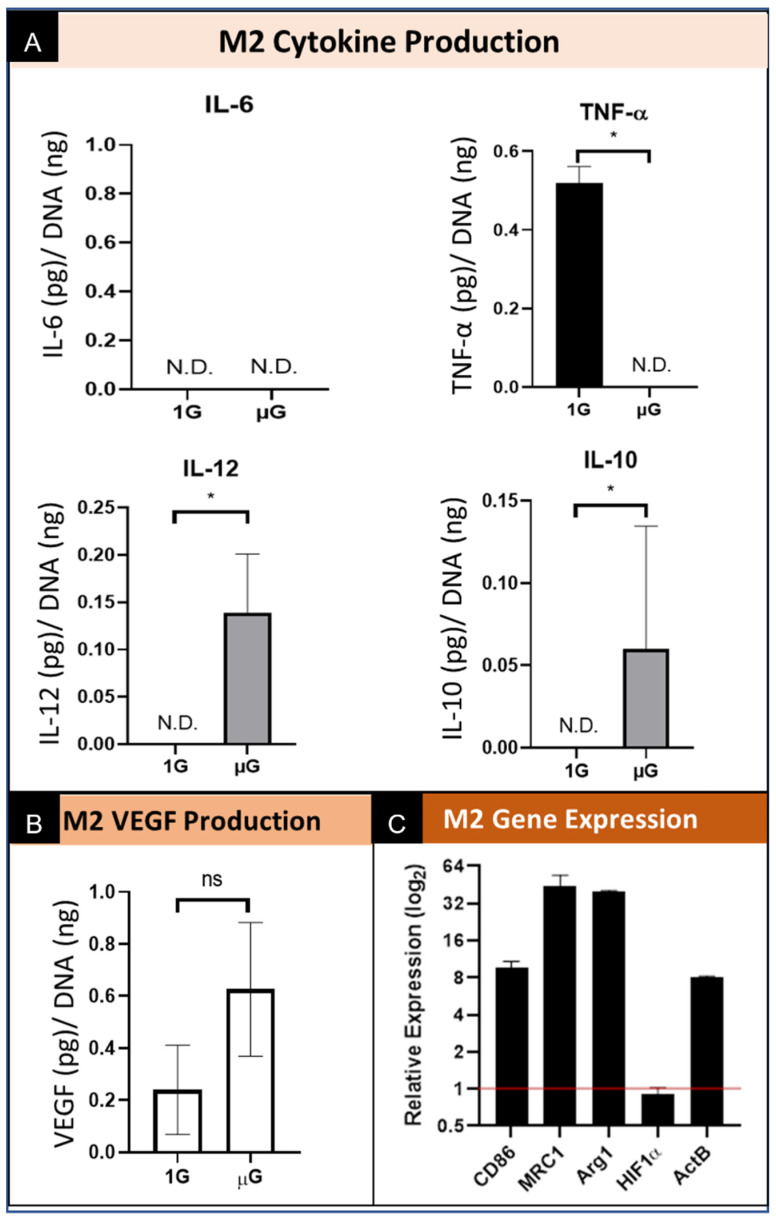
Summary of the effect of simulated microgravity on M2 macrophage protein and gene expression. (**A**) Quantification of M2 macrophage secretion of inflammatory cytokines while cultured in both normal gravity (1G) and simulated microgravity (µG) for IL-6, TNF-α, IL-10, and IL-12. Data normalized to DNA concentration and are presented as Mean +/− SD, *n* = 3. N.D. indicates the concentration did not reach the threshold for the assay and thus is Not Detected. Significance determined by unpaired Student’s *t*-test; * denotes *p* < 0.05. (**B**) Quantification of VEGF secretion by M2 macrophages cultured in normal gravity (1G) and simulated microgravity (µG). Data normalized to DNA concentration and are presented as Mean +/− SD, *n* = 3; Significance determined by unpaired Student’s *t*-test; ns denotes non-significant. (**C**) Gene expression of M2 phenotype macrophages for M1 specific (CD86) and M2 specific (MRC1 and Arg1) genes, HIF1α, and ActB. Data displayed as relative expression of simulated µG samples compared to 1G controls (red line).

Macrophages underwent phenotypic differentiation following stimulation with interleukin 4 (IL-4) into “alternatively activated” M2 macrophages which are pro-healing. Specifically, stimulation with IL-4 drives towards a M2a subtype. Of the other M2 subtypes not directly investigated, immune complexes and LPS can drive toward M2b; glucocorticoids, IL-10, and TGF-β toward M2c; and TLR agonists or IL-6 toward M2d [30,31]. In the macrophages polarized into the M2 phenotype, the level of secreted IL-6 was not detectable from cells cultured in both normal gravity as well as simulated microgravity. (Figure 5A). We also show a decrease in TNF-α secretion to undetectable levels when cultured in simulated microgravity conditions. Conversely, the levels of secreted IL-10 and IL-12 by M2 macrophages cultured in simulated µG was increased over the level secreted by cells cultured in normal (1G) control. As it relates to the angiogenic function, the data show that the M2 phenotype macrophages culture in simulated µG do not have an increase in the amount of VEGF secreted relative to normal 1G control. (Figure 5B) These results demonstrate similar trends in cytokine production and secretion under conditions of microgravity, however we once again see the stimulated secretion of both a pro-inflammatory cytokine (IL-12) and an anti-inflammatory cytokine (IL-10), which again suggests the presence of multiple phenotypes during simulated microgravity culture. These data also suggest that the pro-healing M2 macrophages do not promote VEGF mediated angiogenic stimulation when exposed to simulated microgravity.

#### 3.4.2. M2 Macrophage Gene Expression

M2 macrophages express elevated M1 specific CD86 gene expression under simulated µG compared to the 1G normal gravity controls. Additionally, both M2 phenotypic markers MRC1 and Arg1 were notably elevated in simulated µG. In agreement with the M0 and M1 phenotypes, HIF1α expression did not appear to be meaningfully altered by simulated microgravity, and ActB was increased in simulated µG. In general, simulated µG appeared to stimulate greater phenotypic expression, not only for M2 markers, but for the M1 marker CD86 as well. (Figure 5C) These results demonstrate a strong response by M2 macrophages in the upregulation of M2 specific genes, which maintain the M2 phenotype, however, there is also a concomitant increase in M1 gene expression.

## 4. Discussion

In the present study, we show the individual responses of polarized macrophages (M0, M1 and M2) to simulated microgravity. Our work focusses on changes in both gene expression as well as protein production to better understand the phenotype-specific macrophage response to microgravity. To assess the effect of simulated microgravity across major macrophage phenotypes, we evaluated the expression of key macrophage phenotypic markers. CD86 is a widely used marker of the M1 macrophage phenotype [32,33,34], while Arginase 1 (Arg1) and mannose receptor C-type 1 (MRC1) are markers of the M2 phenotype that have been frequently used in RAW 264.7 cells [18,35,36,37,38,39,40]. CD86 is found on numerous immune cell types including macrophages and provides a co-stimulatory signal for T-cell activation. CD86 has rarely been investigated in the context of macrophage microgravity studies. In our study we found that all three phenotypes of macrophages significantly upregulated CD86 gene expression, with M1 being the most significant. Studies suggest that the increased CD86 expression in antigen-presenting cells in microgravity is affected by signaling via induced MAPK activation [41]. MAPK has been suggested as a secondary signal of microgravity via mechano-sensitive membrane proteins [42]. We also assessed the gene expression for M2 specific genes Arg1 and MRC1. Arg1 gene expression by macrophages is important in mammalian immunology [43]. Arg1 functions by regulating the availability of l-arginine, necessary for nitric oxide production, as well as inhibiting the T-cell mediated immune response. Reports have shown an increase in Arg1 gene expression in primary mouse macrophages under simulated µG via clinorotation following IL-4 stimulation [23]. This result corresponds to the M2 phenotype. In our study, we see an upregulation of Arg1 expression by the M2 macrophages cultured in simulated microgravity relative to normal gravity. The other macrophage phenotypes (M0 and M1) do not show the same upregulation in Arg1 expression. When we consider another M2 marker gene, MRC1, which is involved in both innate and adaptive immune responses, we also see a phenotype-dependent change in MRC1 gene expression, with M2 macrophages showing the greatest upregulation of MRC1 gene expression. Although using a different experimental design, there have been reports of microgravity impairing macrophages polarization, including a decrease in MRC1 when comparing macrophages polarized while under microgravity compared to those polarized in normal gravity [12]. We do see upregulation of M2 specific MRC1 by M1 macrophages, though to a lesser degree. However, as a confounding factor, it is important to note that macrophages phenotypic markers are not absolute, as M1 polarized macrophages can express M2 markers and vice versa [44,45]. Collectively, the gene expression profiling describes a simulated microgravity-mediated shift from the initial M0, M1 and M2 phenotypes to a mixed population of cells exhibiting multiple phenotypes, whereby some cells express M1 and others express M2 phenotypic markers within the same culture. Notably, as several unique subsets of the M2 phenotype have been defined in the literature (M2a, M2b, M2c, and M2d), it is possible that released cytokines induced different phenotyping via paracrine signaling, resulting in mixed expression. In particular, the M2b phenotype can be stimulated via LPS and M2c by IL-10, among other factors [30,31]. The resulting subtypes have different expression profiles, with for instance M2b expressing CD86—considered a M1 marker—while M2a and M2c do not [18]. The M2b subtype also releases pro-inflammatory cytokines, including TNF-α, IL-1β, and IL-6 [46]. Additionally, M2c macrophages are also known as ‘deactivated’ or ‘inactivated’ macrophages, as they have demonstrated deactivation of the M1 phenotype following M1 stimulation and subsequent adoption of the M2 phenotype [47]. Work has been undertaken to describe the complexity associated with macrophage phenotype, for example, macrophages recovering from LPS tolerance are pro-inflammatory while having a distinct regulatory anti-inflammatory profile [48]. There have been calls for increased specificity in nomenclature and characterization regarding macrophage phenotypes, similar to the ranges of activation across T helper cell subtypes, due to indications of more complex macrophage phenotypic behavior [18,49]. The significance of our observed mixed phenotypes on overall macrophage function or dysfunction is an important factor to investigate in understanding the effects of microgravity on specific macrophage activation types.

Like many immune cell types in the body, exposure to microgravity promotes structural changes in macrophages. Previously published macrophage microgravity studies reported on the effects of microgravity on actin and cytoskeleton organization [13,50,51]. Work has shown that cytoskeletal actin arrangement enables both M1 and M2 functionalities [52]. Therefore, we assessed if simulated microgravity affects ActB expression in macrophages. Interestingly, structural changes in macrophages are reported to be acute, occurring within seconds, and are transient. In our study, we found that all of the macrophage phenotypes upregulated actin gene expression when cultured in simulated microgravity. These data warrant further investigation into more detailed cytoskeletal organization and arrangement as an indicator of macrophage function, as a good amount of the existing data remains largely qualitative [53,54]. Additionally, this may indicate the possibility of ActB being a questionable reference gene for genetic expression analysis in the context of microgravity.

Regarding their immunological function, macrophages release several immune-associated cytokines, both pro- and anti-inflammatory. Several major players among these secretory signalers are TNF-α, IL-6, IL-10, and IL-12, which were selected for our study. Several studies have previously investigated macrophage production of these cytokines in the context of microgravity, though reports are across different cell sources and gravity methods, and without investigating these effects based upon macrophage phenotype [5,19,20,21]. Our results show that pro-inflammatory TNF-α secretion by macrophage cells in simulated microgravity was significantly decreased across all three phenotypes evaluated. This agrees with current literature on the downregulation of TNF-α protein expression by macrophages in microgravity [11]. A review of literature highlights the differences in results seen across studies, with some studies reporting increases in TNF-α expression [55,56,57] and others reporting decreases [5,19,20,21] from µG, suggesting that the variety of cell types, microgravity system, duration, and methods used could be contributing factors [10]. Importantly, when compared to studies using the same cell type and microgravity simulation as our study, TNF-α protein expression is largely reported to decrease. In fact, we report complete absence of TNF-α, below the threshold for detection in our system. It has been suggested that the decrease in TNF-α by simulated microgravity may be the results of concomitant increase in the expression of heat shock factor-1 (HSF1) a known repressor of TNF-α promoter [19]. Although consistently decreased, the mechanism by which microgravity exposure affects the expression of TNF-α in macrophages remains to be fully clarified. Nevertheless, these results imply that microgravity exerts a negative impact on the expression of TNF-α in macrophages, and based upon our results, we show that this negative impact is seen in all major macrophage phenotypes.

We also investigated IL-10 and IL-12, which are cytokines produced primarily by antigen-presenting cells, particularly macrophages and dendritic cells. IL-10 and IL-12 are important immunoregulators in host defense and immune homeostasis, with IL-10 being anti- and IL-12 being pro-inflammatory in nature. In our study, all three phenotypes of macrophages showed stimulation of pro-inflammatory IL-12 following culture in simulated microgravity. Interestingly, we also show a significant upregulation of anti-inflammatory cytokine IL-10 by the M1 and M2 macrophages. Although anti-inflammatory, IL-10 induction is frequently simultaneous with the expression of pro-inflammatory cytokines [58]. IL-10 expression can be upregulated through activation of separate extracellular signal-regulated kinase (ERK) and p38 signal transduction pathways [58,59]. This is interesting, because ERK and p38 have been proposed as gravisensitive signal transduction pathways [42]. The ERK and p38 pathways have also been investigated in previous macrophage microgravity literature, with ERK reported to decrease in one spaceflight study based on rt-PCR and whole transcriptomic analysis [12] or remain unchanged in another study of simulated microgravity based on Western blots [23]. The later study also reported activation of the p38 pathway in macrophages from simulated microgravity, which may not be surprising given that p38 are generally considered to be more responsive to stress stimuli including radiation and osmotic shock, while ERK is preferentially activated by growth factors [60]. The reported activation of p38 correlates to the increased IL-12 secretion we report, as p38 activation promotes the induction of IL-12 [61,62] and drives IL-12 production in macrophage inflammatory responses [63,64]. Additionally, ERK signaling mediates negative feedback of the p40 subunit of IL-12 [62] so it is conceivable that microgravity-induced downregulation of ERK signaling could result in heightened IL-12 expression.

Other cytokines, such as IL-6, are involved in both pro- and anti-inflammatory capacities. IL-6 mediates inflammation via inhibition of TNF-α and IL-1, and activation of IL-10. In this case there is disagreement in the literature regarding the effect of µG on IL-6 expression by macrophages. Hsieh et al. reported a decrease in IL-6 [21], while in another study by Wang et al. using primary mouse macrophages in clinorotation, they reported an increase [23]. Additional spaceflight studies using either primary human [5,65,66], mouse [20], or rat cells [56], that only assess cytokine expression profiles in the context of LPS stimulation (i.e., corresponding to M1 polarization), report an increase [20,56] or decrease [5,65,66] in IL-6 production. In yet another study, IL-6 expression is reported to decrease in spaceflight for B6MP102 cell line macrophages without stimulation (which would correspond to our unstimulated M0 phenotype) [22]. In our work we show no significant detectable expression of IL-6 in either normal gravity or simulated microgravity for M0 and M2 phenotypes, and a small yet insignificant upregulation of IL-6 in the M1 phenotype in simulated microgravity. Collectively, this profile of cytokine expression supports what we see in the gene expression profile, which is that when macrophages are synchronized into the three distinctive phenotypes (M0, M1, and M2), then exposed to simulated microgravity, there are phenotypic shifts that result in a mixed population of macrophages and macrophage phenotypes.

Another functionality of macrophages is their involvement in tissue remodeling and vascularization. VEGF is a major stimulator for angiogenesis and a mitogen for endothelial cells secreted by macrophages. To our knowledge, VEGF expression in the context of macrophages in microgravity has not previously been reported. There has been a previous study of peripheral blood mononuclear cells (PBMNCs) that saw an increase in VEGF expression in simulated microgravity, though this is non-specific for macrophages, as PBMNCs constitute T cells, B cells, NK cells, as well as monocytes/macrophages [67]. In general, it is known that VEGF expression by macrophages is stimulated by LPS in a time- and concentration-dependent manner [68], as well as being NF-κB dependent [69]. Additionally, it has been shown that IL-10 can regulate VEGF production in macrophages in a phenotype- and hypoxia-dependent manner [70]. We show significantly increased VEGF secretion by M0 and M1 phenotypes in simulated microgravity, with M2 demonstrating a non-significant upward trend as well. HIF1α is involved in upregulating VEGF under conditions of hypoxia and other stress, however, our data show no change in HIF1α gene expression by any of the macrophage phenotypes cultured in simulated microgravity relative to normal gravity, indicating that it may not be the mechanism by which VEGF is upregulated under these conditions.

## 5. Conclusions

Microgravity is a simulated environment that has demonstrable effects on immunology at both the physiological and cellular level. Understanding the effects this environment has on major immune cell types, such as macrophages, informs our understanding of human health in microgravity as well as cell functionality and behavior. Macrophage phenotypes play a major function within the body, yet previous studies in the context of microgravity have not specifically evaluated M0, M1, and M2 phenotypes. Our data indicate that some aspects of macrophage phenotype expression in simulated microgravity differs compared to normal gravity, while other aspects of the macrophages are similarly affected regardless of phenotype. These changes result in a macrophage population co-expressing M1 and M2 specific genes and secreting pro- and anti- inflammatory cytokines. A better understanding of how populations of macrophage phenotypes are mixed following simulated microgravity could improve our understanding of the effects of microgravity on cell biology and human health.

## Figures and Tables

**Table 1:** Primer sequences for PCR analysis.

Gene Name	Symbol	Phenotype/Function		Rt-PCR Primer Sequence
Cluster of differentiation 86	CD86	M1	Forward	5′-ACGTATTGGAAGGAGATTACAGCT-3′
			Reverse	5′-TCTGTCAGCGTTACTATCCCGC-3′
Mannose receptor C-type 1	MRC1	M2	Forward	5′-GTTCACCTGGAGTGATGGTTCTC-3′
			Reverse	5′-AGGACATGCCAGGGTCACCTTT-3′
Arginase-1	Arg1	M2	Forward	5′-CATTGGCTTGCGAGACGTAGAC-3′
			Reverse	5′-GCTGAAGGTCTCTTCCATCACC-3′
Hypoxia-inducible factor 1-α	HIF1α	Transcription Factor	Forward	5′-CCTGCACTGAATCAAGAGGTTGC-3′
			Reverse	5′-CCATCAGAAGGACTTGCTGGCT-3′
β-actin	ActB	Structure	Forward	5′-CATTGCTGACAGGATGCAGAAGG-3′
			Reverse	5′-TGCTGGAAGGTGGACAGTGAGG-3′
Glyceraldehyde-3-phosphate dehydrogenase	GAPDH	Housekeeping	Forward	5′-CATCACTGCCACCCAGAAGACTG-3′
			Reverse	5′-ATGCCAGTGAGCTTCCCGTTCAG-3′

## Data Availability

Data are contained within the article.
